# Aggregated Distribution as an Explanation for the Paradox of Plankton and Collective Animal Behavior

**DOI:** 10.3390/biology11101477

**Published:** 2022-10-09

**Authors:** Javier Falgueras-Cano, Juan Antonio Falgueras-Cano, Andrés Moya

**Affiliations:** 1 Institute for Integrative Systems Biology (I2SysBio), University of Valencia and CSIC, 46980 Valencia, Spain; 2 Department of Languages and Computer Science, University of Málaga, 29016 Málaga, Spain; 3 Genomics and Health Area, Foundation for the Promotion of Sanitary and Biomedical Research (FISABIO), 46020 Valencia, Spain; 4 Biomedical Research Center Network of Epidemiology and Public Health (CIBEResp), 28029 Madrid, Spain

**Keywords:** multilevel selection, cellular automata, evolutionary cellular automaton, small-portion effect

## Abstract

**Simple Summary:**

*ECA* is similar to a cellular automaton that mimics the evolutionary dynamics of species in metapopulations, simulating the underlying mechanisms of natural selection. In this work, we carry out an in-silico study of the effects of different dispersal strategies on the evolutionary balance of interactions between digital organisms. In *ECA* we see how a specific type of distribution can significantly influence the dynamics, persistence, distribution, and abundance of populations of different species within a particular habitat. In the first place, we show that an aggregate distribution is more inefficient than a uniform distribution. Still, we verify that this aggregate distribution is essential in predator–prey type interactions so that the species involved do not become extinct. We also show that the aggregate distribution does not comply with the competitive exclusion principle and, for this reason, it results in a general and straightforward explanation for the paradox of plankton and the grouping of animals. Many animals and especially some planktonic species group together in specific spaces, although some travel adrift, leaving other areas or patches free where competitors or their prey can prosper, preventing them from becoming extinct.

**Abstract:**

This work analyzes the evolutionary consequences of different aggregation levels of species distribution with an Evolutionary Cellular Automaton (*ECA*). We have found that in habitats with the same carrying capacity, aggregated distributions preserve smaller populations than do uniform distributions, i.e., they are less efficient. Nonetheless, we have also found that aggregated distributions, among other factors, can help the evolutionary stability of some biological interactions, such as predator–prey interactions, despite their granting less individual fitness. Besides, the competitive exclusion principle does not usually stand in populations with aggregated distribution. We have applied *ECA* to study the effects of aggregated distribution in two notorious cases: in the so-called *paradox of the plankton* and in gregarious animals. In doing so, we intend to ratify long-established ecological knowledge explaining these phenomena from a new perspective. In the first case, due to aggregate distribution, large aggregations of digital organisms mimicking very abundant planktonic species, leave large patches or oceanic areas free for other less competitive organisms, which mimic rare species, to prosper. In this case, we can see how effects, such as ecological drift and the small portion, act simultaneously. In the second case of aggregation, the aggregate distribution of gregarious animals could be explained under specialized predator–prey interactions and interdemic competition. Thus, digital organisms that imitate predators reduce the competitive capacity of their prey, destabilizing their competitiveness against other species. The specialized predator also goes extinct if the prey goes extinct by natural selection. Predators that have an aggregate distribution compensate the prey and thus avoid exclusion. This way there are more predator-free patches in which the prey can prosper. However, by granting greater colonization capacity to its prey, the predator loses competitiveness. Therefore, it is a multilevel selection event in which group adaptation grows to the detriment of the predator as an individual.

## 1. Introduction

The ecological distribution of populations describes the spatial location of organisms. The distribution depends on intrinsic factors derived from the biology of each species and on extrinsic, i.e., biotic and abiotic, factors of the environment in which they live [1]. In ecology, dispersion of an organism refers to its permanent movement outside its origin and long-term settlement in a new location [2]. The spatial pattern of the individuals within the same population defines their distribution as clumped, uniform, or random. The nearest neighbor ratio and the range of separation from the parents also show either local or global distributions. Spatial pattern, how it arises and how it is maintained, are central foci of ecological theory [3]. Some theoretical models use different parametric formulas to define dispersion strategies depending on the *dispersion nucleus* (i.e., the probability function that describes the probability of dispersion at different distances [4,5,6,7]). They are mostly integro-differential equations applied to the dispersion itself [5] or to the introduction of invasive species [8].

Dispersion is a phenomenon studied at length in meta-population dynamics, given its contribution to colonization capacity [9,10]. Yet, little is known about whether the different dispersion strategies can generate population distributions affecting the fitness of organisms [2]. Ecological and evolutionary literature has widely focused on the effects of distributions [11,12]. However, there has been a much less focus on the particular dispersion strategies, maybe because of the notorious experimental difficulty to prove the proposed mechanisms that affect the evolution of dispersion [13]. There is a gap between the theories related to population distribution and their experimental studies, which logically affects our understanding of why particular organisms disperse in a certain way. Sometimes, there is no clear theoretical basis in biology on which to test hypotheses about the dispersal of specific populations. Among others, biogeography [14], collective animal behavioral studies [15], or conservation literature [16] focus on measuring population densities and their influence on variables, such as spatial distribution and its influence on species conservation, and also address studies on collective animal behavior. In contrast, evolutionary ecology focuses on studies based on the degree of local adaptation [17]. Much progress has been made in the use of techniques that measure the identification of aggregation or dispersion, even at various scales [18].

In this work, we undertake an in-silico study into the effects of the different dispersion strategies on the evolutionary equilibrium of interactions between digital organisms. In *ECA*, a specific type of distribution can strongly influence the evolutionary structure of a given community, its dynamics, persistence, distribution, and the population abundance of different species within a specific habitat.

## 2. Materials and Methods

### 2.1. About ECA

*ECA* stands for *Evolutionary Cellular Automaton* [19]. *ECA* starts from a data set obtained from natural observations or lab experiments indicated in each case or introduced by the user without reference to empirical studies. Such data constitute the configuration or initial status of each simulation. *ECA* is not a Cellular Automata or an Evolutionary Algorithm, although it has similarities. We are specifically interested in simulating diverse types of dispersion in different contexts to study their evolutionary consequences.

Terminology and concepts involving *ECAs* are provided in detail in [19]; notwithstanding, we focus on some of the most relevant terms related to dispersion. First of all, the *habitat variables* that define the selective pressure:

- *NumberOfCells:* number of cells in which the habitat is divided.
- *NumberOfRsrcsInEachCell:* discrete quantity of resources in each cell.
- *Distribution:* type of structural distribution of the populations in the habitat.

Secondly, we take into account the *species-related variables* that define the biological efficiency of species:

- *id*: name of the species.
- *NumberOfItems:* size of the initial population.
- *DirectOffspring:* number of direct offspring of each species.
- *Distribution*: functional distribution of the species or *vagility*. If this variable is not defined for a species, the program selects the variable *Distribution* of the habitat variables by default.
- *IndirectOffspring:* number of indirect offspring.

In *ECA*, digital organisms compete in each of the cells for limited resources (“*NumberOfRsrcsInEachCell*”). Only those that access the resources or means can reproduce. Following the Hamilton theory [20], the number of offspring depends on its direct capacity to replicate (“*DirectOffspring*”), if it is not associated, and also on the indirect (“*IndirectOffspring*”) if it is associated with another organism. Emulating the theory of multilevel selection [21], the organisms can also be grouped forming a new and more complex organism, different from their grouped ones. At each discrete step or generation, the descendants are dispersed throughout the cell lattice (“*NumberOfCells*”) according to dispersion strategies (“*Distribution*”).

### 2.2. Dispersion Strategies

We focus not only on the effects of the nucleus and the dispersion distance over the structure and evolution of metapopulations but also on assessing whether the different types of dispersion affect the fitness of organisms and species evolution, or on the contrary, we must assume the neutral theory, which considers that the dispersion is stochastic and similar among species [2].

In *ECA*, the digital organism is considered as an instance, and its dispersion strategy as an emergent property. The distribution derived from the starting variables of the initial configuration can be more or less uniform, local or global. Thus, a population or species follows a strategy of aggregated distribution when the offspring is distributed in the cells with greater variability than the mean, i.e., with greater variance. We propose two strategies (*n* or *r*) that the user, as a person using the program, can choose in the initial configuration. A prefix designates the degree of dispersion to *n* and *r*. Such a prefix ranges between 0 and 100 and represents a percentage.

**Figure 1:**
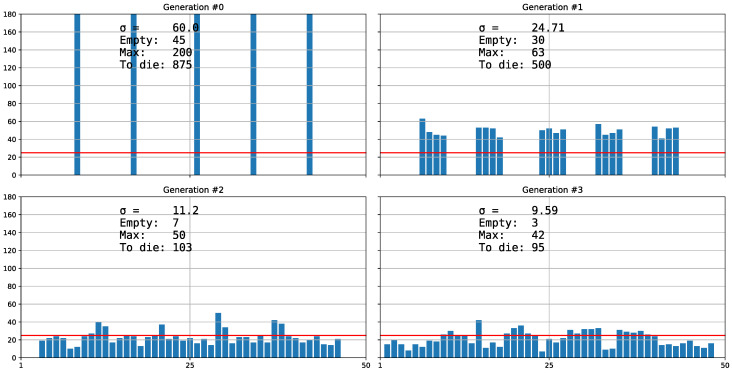
Strategy *n*. The *Y*-axis represents the species population, and the *X*-axis the 50 cells (*nCells* in the figure). In the first chart or Generation #0, the 1000 organisms distribute themselves among five equidistant cells out of the 50 that are available. The following charts depict how in the next generations, organisms distribute themselves randomly within their neighboring cells, one by one, under the distribution *6n*, which equates to distribution among the three adjacent cells on both sides of the cell of origin. Observe that the greater the iteration of generations, the greater the uniformity of distribution, the lesser the variance (σ in the figure), the lesser the population density in the most populated cell (Max in the figure) and the greater the number of organisms that die because they cannot access the limited resources (*To die* in the figure). In this example, the carrying capacity is *k* = 25 (red line in the figure), meaning 25 organisms can survive in each cell.

The *n* strategy (*neighbors_distribution*) would usually generate a local distribution. From one generation to the next, the offspring distribute themselves at random within the neighboring cells, one by one. The user selects the range of adjacent cells (or *neighborhood ratio*) at the initial configuration *(Distribution)*, from only one cell (named *0n*) under which the offspring would stay in their cell of origin, to all the cells (*100n*). On increasing the neighboring ratio and the number of generations, the uniformity of distribution also increases (Figure 1).

**Figure 2:**
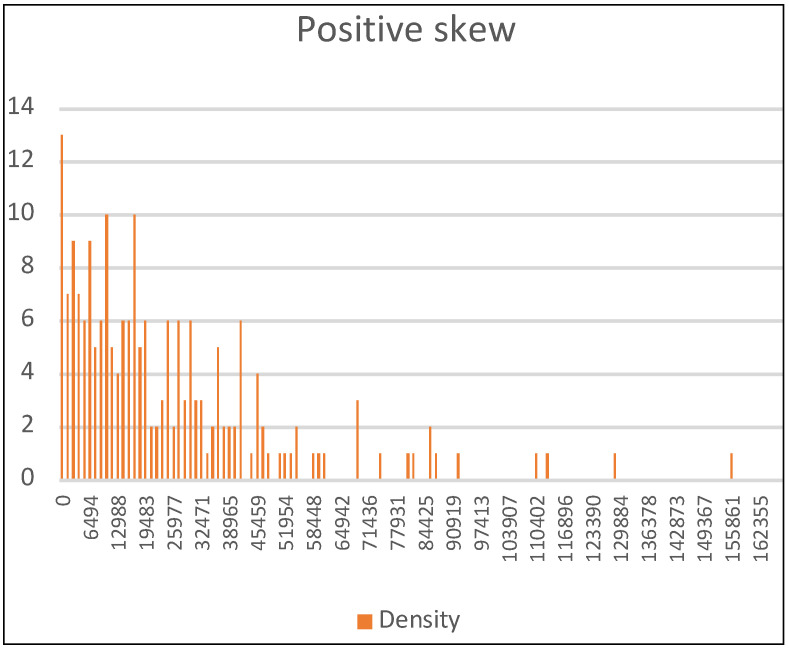
We represent a distribution generated by *ECA* for *Distribution* = *100r* of a population of 5,000,000 organisms. The *X*-axis represents the density of each cell and the *Y*-axis is the number of cells. The skewness coefficient is positive (2.08) and the standard deviation is 24,738.08. It is a negative binomial distribution.

The *r* strategy (*random_global_Avg*) generates a global distribution where the total number of organisms disperses globally in groups of random size without considering the cell of origin. The density of each cell is calculated with a list of random numbers ordered from the smallest to largest. Afterwards, each random number is subtracted from its previous number. We have verified that this list of random differences generates an aggregated distribution with a positive skew, in which very few cells have high density, and many others are not very dense. The distribution is non-parametric and well fitted to the traditionally recommended functions [22]. It is similar to the negative binomial distribution (Figure 2), which has been proposed as a starting point for quantifying and modeling count data in studies on ecology and biodiversity in cases of overdispersion or aggregation [23].

**Figure 3:**
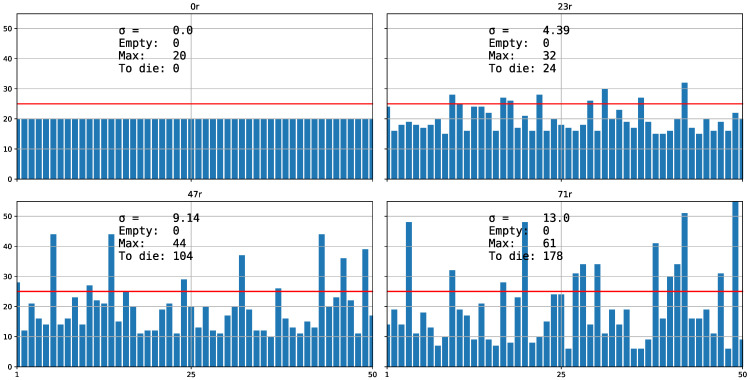
Strategy *r.* In this case, each chart is not a successive generation as shown in Figure 1. Each chart represents a different distribution strategy, which successively increases the prefix assigned to *Distribution* in the initial configuration by 23 percentage points (from *0r* to *73r*). The higher the assigned prefix, the more aggregated the distribution. The greater the variance (σ in the figure), the higher the density of the most populated cell (*Max* in the figure) and the greater the number of excluded organisms (*To die* in the figure) because they exceed the carrying capacity (*k* = 25, line red in figure). There are no empty cells (*Empty* = 0 in the figure). The *h* strategy is a variant of the *r* strategy in which the user chooses the proportion of the dispersion reticulum reduction, with the rest of the cells being empty (Figure 4).

The dispersion range varies from a maximum (*100r*) with maximum variance to a minimum (*0r*) with minimum variance (Figure 3). In this case, the suffix of *r* indicates the percentage at which the random variable approaches the average: the smaller the suffix, the closer the variable is to the average and, therefore, there is less variance.

Following these strategies, we can obtain a local aggregated distribution (maximum variance, *0n*) or more uniform local distribution (maximum uniformity, *100n*) but also a global aggregated distribution (maximum variance, *100r*) or more uniform distribution (maximum uniformity, *0r*). Strategies *100n* and *0r* practically distribute the average in each cell. Strategy *h* represents the organisms with innate mechanisms that make them seek out their conspecifics for grouping. The more aggregation there is, the more there are vacant cells.

### 2.3. Software

We have used free software (Python version 3.7, Wilmington, DE, USA) with numerical libraries numpy and graphics matplotlib and gnuplot and the utilities of the operating system UNIX in OSX. We have also used Excel (Redmond, WA, USA) for intermediary tests. Some simulations were run in the Picasso supercomputer at the SCBI (University of Málaga, Málaga, Spain). The program can be accessed at https://github.com/juanfal/AE4 accessed on 15 July 2022.

## 3. Results and Discussion

### 3.1. The Inefficiency of Aggregate Distribution

**Figure 4:**
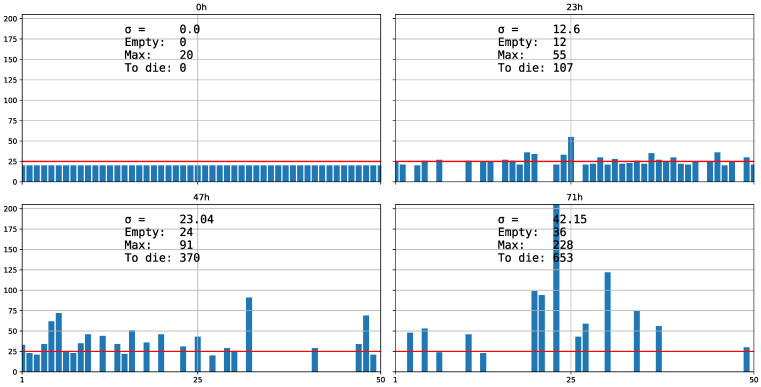
Strategy *h*. It is similar to *r* distribution (Figure 2) but with the percentage of empty cells that its prefix indicates (Empty in the figure).

In the study of the predator–prey equilibrium, we observe that *Neodiprion sertifer* reaches stability at 12,000 organisms when there is a more uniform distribution (*100n*), whereas with an aggregated distribution (*100r*), only 9000 survived, considering a carrying capacity of 100 cells times 120 of “*NumberOfRsrcsInEachCell*” and regardless of how we define *N. serfiferous*, because they are relative comparisons. We see that the difference comes only from the variance (simulations 1 and 2 in *1Pred.json* in Supplementary Material) [24]. We also observe that for the three types of dispersion strategies (*n*, *r* and *h*; Figure 1, Figure 3 and Figure 4), when the standard deviation σ in the distribution is higher, more organisms are excluded (or more die). We will check whether the greater the variance in the distribution, that is, the more aggregate it is, the more inefficient it is, because fewer organisms survive for the same potential carrying capacity.

Chebyshev’s inequality [22] also allows a probabilistic result indicating that aggregated distribution is more inefficient. Such inequality reveals the ceiling or threshold for the probability that a random variable *X* with a finite variance σ is placed further than distance α from its average μ:

(1) $$P\left(\left|X-\mu \right|\geq \alpha \right)\leq \frac{\sigma^{2}}{\alpha^{2}}\;\;\;\;$$

Equation (1) gives us the area under the density curve, if the variable is continuous; or, if the variable is discrete, it provides the sum of the corresponding probabilities of the two extremes farthest from the mean μ:

(2) $$P\left(X\geq \mu +\alpha \right)+P\left(X\leq \mu -\alpha \right)\leq \frac{\sigma^{2}}{\alpha^{2}}\;\;\;$$

If the distribution is symmetric towards its average μ, without skew, the two summands of (2) are the same, so:

(3) $$P\left(X\leq \mu -\alpha \right)\leq \frac{1}{2}\frac{\sigma^{2}}{\alpha^{2}}\;\;$$

However, if the distribution is skewed with positive or negative skew, (3) it would result in:

(4) $$P\left(X\leq \mu -\alpha \right)\leq q\frac{\sigma^{2}}{\alpha^{2}}\;\;$$

where in q≥0, because it is a proportion between two areas of a density distribution or between sums of probabilities. For our study in *ECA*, if we named the average carrying capacity of the habitat *k* and we considered the probability of the variable *X* as the probability of an organism to be allocated in a cell with a certain amount of resources, we can replace α=μ−κ in (4):

(5) $$P\left(X\leq \kappa \right)\leq q\frac{\sigma^{2}}{{\left(\mu -\kappa \right)}^{2}}\;$$

That would be the maximum probability level that an organism is excluded from reproduction because the population exceeds the carrying capacity. We see that the ceiling (Equation (5)) is directly proportional to $\sigma^{2}$. This implies that when the distribution becomes more aggregated with more deviations from the average, such a distribution becomes more inefficient because it increases the probability ceiling of an organism reaching a patch where the population density is so great that resources are insufficient and it is excluded. Furthermore, as we observe in Equation (5), the degree of such inefficiency can increase if *k* approaches μ or if factor *q*, which is related to distribution skewness, increases. On the other hand, the correlation between inefficiency and $\sigma^{2}$ in (5) may be very small when $\left(\mu -\kappa \right)$ is large; in other words, when there is too much or too little selective pressure, or when distribution skewness makes “*q*” have a small value.

### 3.2. Aggregate Distribution Stabilizes Predator–Prey Interactions

It is known that a nonlinear predator functional response depending on local prey density can induce the formation of static patterns in prey density and thus lead to stable local and global dynamics of the interaction [25]. With *ECA*, we also see that the aggregated distribution is the only configuration that allows for evolutionarily stable strategies in biological interactions in some contexts, such as between the cuckoo (*Cuculus canorus*) and the reed warbler (*Rocephalus arundinaceus*) [26,27]. The cuckoo places its egg in the reed warbler’s nest so that the latter raises it as its own. In *ECA*, the cuckoo has a *DirectOffspring* and an *IndirectOffspring* of 0 and −2, respectively, and the warbler of 2 and 2 so that when they do not interact because they do not exchange eggs in a cell, the reed warbler has two chicks, whereas if they interact and the cuckoo exchanges eggs, only the cuckoo will have two chicks. Regardless of the number of chicks they have and the other parameters defining species in the initial configuration of the simulation, when one of the two populations maintains a strategy of local *100n* with low standard deviations, the cuckoo becomes extinct (simulation 1 and 2 in *1Para.json* in Supplementary Material). Both species survive only under a strategy *100r* with very high standard deviations (simulation 3 in *1Para.json* in Supplementary Material). For this to happen, the habitat has to be highly fragmented (1000 cells in this case); if it is reduced to 100 or 10 cells (simulations 4 and 5 in *1Para.json* in Supplementary Material), both populations will suffer from fluctuations to the brink of extinction [28,29,30]

### 3.3. Aggregated Distribution: Another Explanation for the Paradox of the Plankton

The principle of competitive exclusion [24,25] states that two species in competition cannot coexist in the same habitat and niche and the organism with greater fitness will prevail. This theory has been supported by mathematical models based on competition, such as Lotka-Volterra’s, and several investigations into experimental evolution carried out in chemostats [31,32,33]. Nonetheless, many competitive species that coexist in natural aquatic ecosystems stand against this theory [34,35]. This contradiction is known as *the paradox of the plankton* [36], and it has been resolved by adding extrinsic factors such as global warming, immigration, latency, special heterogeneity of habitats, and chaotic dynamics [37,38,39]. Some of these factors are difficult to apply to aquatic ecosystems, characterized by their homogeneity and by hosting a large number of species in competition. Yet, as we will see, trophic interactions can also generate spatial heterogeneities [40,41].

**Figure 5:**
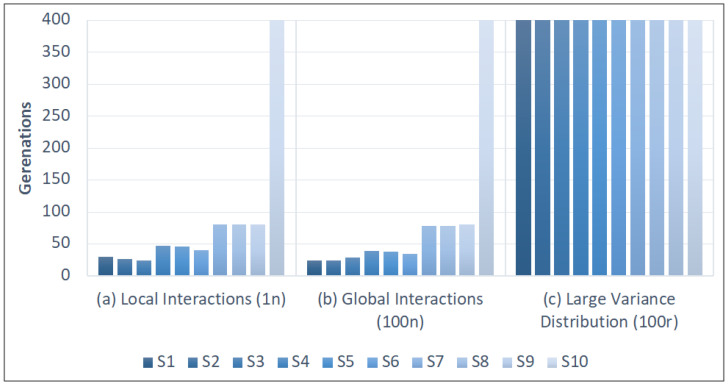
Local vs. global interactions. Data obtained from *1Plank4.json* in Supplementary Material: 10 species (*S1* to *S10*) compete on equal terms, with only variations in the descendants (*S1*–*S3* have 3 *DirectOffspring*, *S4*–*S6* have 4, *S7*–*S9* have 5, and *S10* have 6 *DirectOffspring*). We use a different dispersion strategy in each figure. Each blue bar on the X -axis represents the surviving generations of each of the 10 species until each becomes extinct by competitive exclusion. We observe that the more competitive species remain longer when they interact locally under the strategy *1n* (**b**) than when they use the strategy *100n* (**a**). This difference is more significant with aggregated distribution, which has a greater variance, such as *100r* (**c**): no species is excluded in 400 generations.

Another explanation stems from intrinsic factors, such as local interactions and the finite ecological time scale [42]. We can test this thesis in *ECA* by considering the distribution strategy *1n*, which implies a random offspring distribution but only in the adjacent cells. We thus observe that local interactions indeed prolong the coevolution of competitive species (simulations 1 and 2 in *1Plank4.json* in Supplementary Material) in comparison with global exchanges. A distribution strategy of *1n* implies that organisms stay closer to their parent’s cell whereas offspring are distributed among all cells in a more global strategy (of *100n*). Therefore, *1n* produces more uneven-sized groups with more deviations from the average (Figure 5a). What happens if the distribution has more variance? For example, the maximum *100r* (see simulation 3 in *1Plank4.json* in Supplementary Material). We observe that all species remain in coevolution under stable equilibrium after 400 generations, despite their different fitness (between 3 and 6 descendants on average) with a general population proportional to the fitness of each (Figure 5c). These results show that aggregated distribution explains the paradox better than local interaction.

A global and aggregated distribution such as *100r* can be more convincing in habitats, such as oceans. They represent the most extensive continuous environment on Earth. With regards to marine plankton and the communities of small pelagic species, despite being sessile and of free life, it seems that body size, local abundance and oceanic drifts are key factors in the global biodiversity patterns. It is, therefore, likely that communities of tiny organisms (body size < 2 mm) and with high local abundance have a global panmictic distribution [43]. Certain environmental conditions, such as water temperature, salinity, luminosity, vertical water movements or the availability of nutrients cause local plankton concentrations to form colonies or aggregate in suspension, until reaching extreme flowering levels (or *Bloom*) that expand it explosively [44]. This indicates that plankton tends to group to a large degree together despite its great mobility throughout marine currents. In recent decades, several satellite algorithms have been proposed to recover information on phytoplankton groups using ocean color data [38] that can identify key groups and study their spatial-time distribution [45,46] They all rely on the premise that the most abundant key groups of plankton are distributed by grouping, or they would not be detected. Other plankton species that tend not to aggregate have a more uniform distribution and are, therefore, not considered here. By contrast, nekton could be considered, as it does tend to aggregate due to trophic interactions.

**Figure 6:**
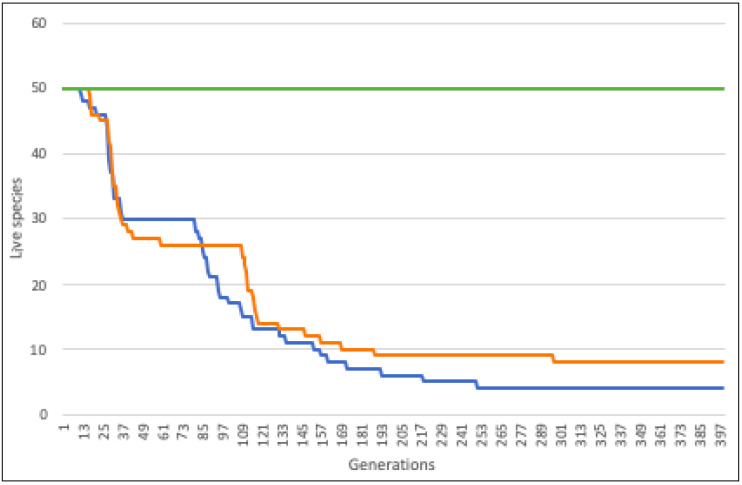
The paradox of the plankton at large-scale. Data obtained from *2Planck1.json* in Supplementary Material. The *X*-axis represents the 400 generations and the *Y*-axis the species that coexist despite all of the 50 species having different fitness. The fitness grows from *S1* in which *DirectOffspring* is 10, to *S50* in which *DirectOffspring* is 59. When the distribution is uniform globally (blue and orange curves with strategies of *1r* and *20r*, respectively) the species with lower fitness become progressively extinct; whereas when the distribution is aggregated, with more variance (the green line represents the distributions of *40r*, *60r*, 80r and *100r*) all the species remain in equilibrium; none is excluded even though species *S1* had 10 descendants on average and *S50* had 59.

To scale the simulation to more realistic situations, we have used a supercomputer that has considered 50 populations of 50 species with different fitness (correlatively from 10 to 59 of *DirectOffspring*), species that compete in a habitat with a carrying capacity of more than 800 million organisms (see *2Planck1.json* in Supplementary Material). We have only modified the global dispersion strategies in each simulation, from more (*1r*) to less (*100r*) uniformity. We have observed that: (a) when the variance is greater, a larger number of species coexist for longer (Figure 6); (b) the population size of each species depends significantly on its fitness (simulations 1 and 2 in *2Planck1.json* in Supplementary Material); and finally, (c) the greater the variance in the distribution, the lesser the differences between the general densities per species.

Rare phylotypes coexist within the marine plankton with more abundant species in evolutionary equilibrium. They are in aggregated groups of different densities, which corresponds closely to what we obtain in our model. Our model does not allow the limitless coexistence of species. However, aggregated distribution may be possible because the species with lower population density will be excluded due to ecological drift (simulation 1 in *2Plank2.json* in Supplementary Material). Other models, such as those representing spatial heterogeneity [47], multiple-resource competition, or the interaction between competition and colonization [8,9], may provide a partial explanation for the exception to the principle of competitive exclusion by increasing the size of the system; however, they cannot explain the plankton paradox due to the homogeneity of marine resources. Precisely, due to the grouping of species, this possible homogeneity at the oceanic scale is broken by the increase in the density of resources by absorption or by the formation of marine snow. Besides, they allow the theoretical coexistence of an unlimited number of species, whereas species are limited in marine systems. They are complex models because they require multiple factors or limiting resources and diverse capacities in competition and colonization in the species and the compensation of local and partial imbalances to achieve a stable general equilibrium, and still, such equilibrium could be more unstable than that observed in marine ecosystems.

Similarly to competition models analyzing the classic Lotka-Volterra equation, others reveal that the coexistence of two or more species is only possible if the intraspecific competition is stronger than the interspecific competition [48]. That is why the effect of density under traditional mathematical models of population dynamics is treated either linearly or as a constant, with an *average overcrowding* [49,50,51,52,53]. These models depict the coexistence of a few species, not many. Others, such as the modified Lotka-Volterra competition model of Taylor and Crizerque [54], consider the effect of non-linear overcrowding or assume that the mortality rate of an individual increases with population density (non-linear) [55], while others introduce variables such as immigration [56]. These models can demonstrate the coexistence of species, but only in local situations and particular circumstances of equilibrium.

**Figure 7:**
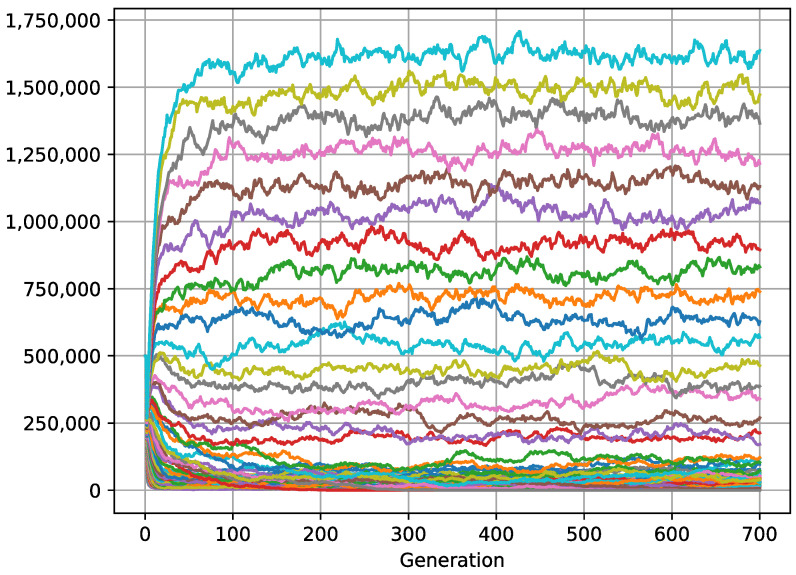
Data obtained from *2Plank2.json* in Supplementary Material. A total of 100 species with aggregated distribution compete in a habitat of 20,000 cells. Each species is represented by a color. After 700 generations, 92 species still coexist in stable evolutionary equilibrium, while only 55 species managed to coexist under the same conditions and in the same generation but with 12,650 cells (see simulation 1 of *2Plank2.json* in Supplementary Material). More fragmentation increases the probability of ending up in cells where a few competent organisms concur.

Unlike the above, our model does not restrain the underlying mechanisms inherent to natural selection to parametric functions. It achieves the coexistence of multiple species with a variable density that significantly depends on the level of competition. The greatest biodiversity is generated by a sole ubiquitous factor: the standard deviation of the distribution. Irrespective of inherent variables particular to each species, the more variance in the distribution and habitat fragmentation (Figure 7), the more species coexist. It also combines other effects, such as ecological drift or the *small portion effect* (simulation 1–2 in *2Plank2.json* in Supplementary Material) under which the smaller species have the opportunity to eat the leftovers of the larger. The species with higher fitness are majoritarian, but they cannot access the leftover resources when they represent a fraction of what they need to survive, while those with lower fitness are able to access the small portions. Due to this small-portion effect, species that have intermediate fitness become extinct.

Our explanation is simple and general. In a cell or patch, randomness in dispersion variability can make a small portion of very competent species concur with a large proportion of less competent species, giving the latter a better chance of accessing resources and reproducing. The compensation that would be obtained in competition/colonization in the traditional differential model is exercised here by competition in density, and it only requires variance increase in the dispersion strategy. For species with low fitness that are not very competitive, these patches are true adaptation refuges that allow them to repopulate the habitat. The big groups of the most abundant plankton species vacate these marine zones for rare phylotypes to proliferate.

Due to aggregated distribution, these adaptation refuges or competition refuges also appear in the case of rainforest dung beetles [57]: an aggregated distribution of manure and the natural variability in the size of the patch contribute to the coexistence of species because they create low density refuges for weaker competitors.

### 3.4. An Explanation for Animal Aggregation

The aggregation effect or overcrowding is considered as one of the most ubiquitous mechanisms in any biological population [49,50,51,52,53,58,59,60,61]. Flocks of birds, ungulate herds, schools of fish, swarms of Antarctic krill, pods of dolphins, or swarms of red locusts are examples of animals’ collective behavior. Such behavior, because it occurs in such diverse organisms, in each case has been justified by its adaptive social and genetic function, anti-predator strategy, feeding optimization, or increased efficiency in locomotion [62], population viscosity is generally beneficial to cooperation, because cooperators can obtain additional benefits by being grouped together [63].But these are mere extrinsic functions, as they cannot be extended to other scenarios or general situations. Besides, they do not explain the grouping tendency in other organisms, such as in groups of bacteria in which bacteria stay together after cell division or even move collectively creating dynamic bacterial groups akin to other systems that show a polarized collective movement, such as flocks of birds or schools of fish [64]. Animal groups present advantages in some situations [62,64,65,66,67,68,69,70,71,72,73,74,75,76,77,78,79], but they can also have important negative repercussions such as raised levels of ectoparasites and pathogens [80,81,82], or increased stress due to the scarcity of resources and reproduction problems caused by higher intraspecific competition [83,84,85,86,87,88,89,90]. This suggests that the biological profitability of animal grouping should be examined on a case-by-case basis.

Other than gregarious behavior, we know that species can reduce their risk of extinction by so-called *adaptive rescue*, characterized by the nature of its distribution strategies [91]. These strategies not only reduce the risk of stochastic extinction [92]; if a population extinguishes by chance, recolonization is more likely if the dispersion rate is high [93]. In arid ecosystems, local facilitation is an essential process to drive bistability and vegetation patches [94]. We know that greater dispersion means more colonization capacity, which could compensate for a smaller competition capacity [10,95]. We could consider that the greatest dispersion in any distribution is achieved not only by reaching the most remote locations, but also by grouping individuals, relocating them from the less dense locations to the more populated. This consideration could imply that having an aggregated distribution translates into an intrinsic adaptive advantage that is ubiquitous in natural selection, although more biologically inefficient, as previously stated.

The aforementioned consideration has been verified in *ECA.* We have observed that a species with a more uniform distribution excludes other species that have an aggregated distribution, even when the number of descendants and the other parameters are equal (see *2Fitness.json* in Supplementary Material). This result has also been verified when the carrying capacity is not uniform in every cell (*NumberOfRsrcsInEachCell*), but variable. To do so, we have artificially grouped two species, *A* and *B,* which only feed and reproduce when grouping with a third species we named *Resources*. *Resources* can have either an aggregated (*100r*) or uniform (*100n*) distribution. Still, in either case, species *B*, which has a more uniform distribution (*100n*), always ends up excluding *A*, which has an aggregated distribution (*100r*), even if both have identical offspring (see simulations 5 and 6 in *2ResV.json* in Supplementary Material). If both have the same uniform distribution strategy but one has a higher number of offspring, the organism with fewer offspring is excluded under the principle of competitive exclusion, irrespective of the variability of the *Resources* (simulations 1 and 2 in *2ResV.json* in Supplementary Material). If we configure species A with more offspring (*DirectOffspring* 3 of *A* against 2 of *B*) and with a less uniform distribution than *B* (*100r* against *55r*), they both remain in equilibrium and do not exclude each other. This result also happens irrespective of the variability of the *Resources* (simulations 3 and 4 in *2ResV.json* in Supplementary Material). Species *A*, with aggregated distribution, gives an advantage to species *B*, as the cells occupied less by *A* allow *B* to compensate for the disadvantage of its lower number of offspring. *A* has more offspring, but affords *B* colonization capacity, and does not exclude it, which is why the principle of competitive exclusion is broken. In *ECA* from a new perspective, we have been able to verify this phenomenon, which is well known and is supported not only by modeling but also by empirical work regarding multilevel selection.

**Figure 8:**
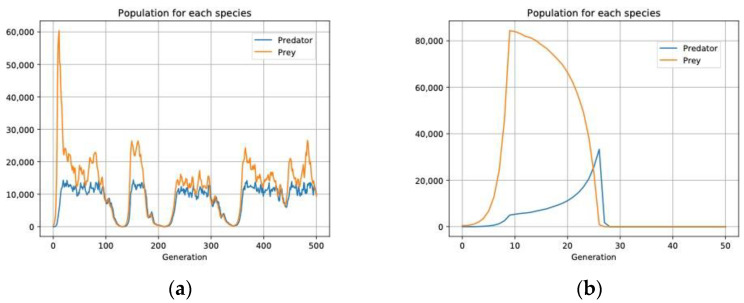
The predator only has offspring if it hunts its prey and kills it. The prey has offspring if it is not hunted. The interaction remains in evolutionary equilibrium if both species have an aggregate distribution (*100r*) as in (**a**). If the distribution is uniform (*100n*), as in (**b**) the interaction barely lasts for 30 generations.

In *ECA* we have also verified that the predator–prey interaction remains in evolutionary equilibrium if there is an aggregate distribution for both species (Figure 8a). But if the distribution is uniform (Figure 8b) either the prey collapse or the predator becomes extinct.

As in the Lotka-Volterra predator–prey equation [10,95] and the Nicholson–Bailey model [96,97,98] we observe the different types of results and how the prey and predator populations are mutually self-controlled without changing any parameters in the equation, but just the distribution strategies [99]. Both predator and prey must follow an aggregated distribution strategy (of *100r* both) for the equilibrium situation to endure (simulation 3 in *2Predat.json* in Supplementary Material). In *ECA* we see that with an aggregate distribution in each cell there is more variability in the density of both species, this makes it easier for few predators to coincide with many prey in a greater number of cells, and thus the prey have a greater opportunity to repopulate the habitat.

As we previously mentioned, there are animals with innate mechanisms to seek their conspecific for grouping in natural situations: they are gregarious animals that should at least have the quorum sensing capacity to detect and respond to the population density [100,101]. These gregarious animals depend more on their intrinsic functionality or vagility to group than on the structural permeability or connectivity of the habitat. We have proposed the distribution strategy *h*, as a subtype of *r* global upon which descendants distribute themselves among some cells, leaving the rest empty. This type of strategy implies a quorum detection capacity to leave cells empty and adopt more aggregated distribution, as groups become bigger. That is why *h* is closer to representing gregarious animals that actively flee from the unpopulated locations.

### 3.5. Interdemic Competition in Multilevel Selection Theory

**Figure 9:**
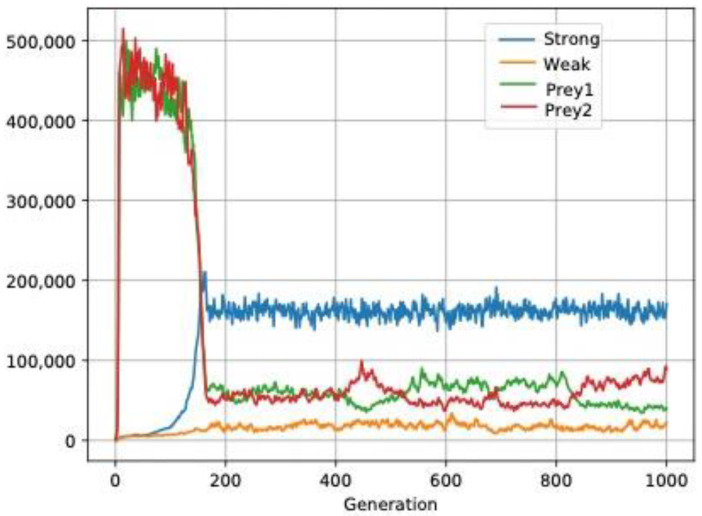
Generalist predators remain in equilibrium: two generalist predators and their respective prey. Strong is more efficient because it has a more uniform distribution. Although at the beginning there is a larger prey population, the dominant predator ends up being majoritarian. Neither becomes extinct.

We have also analyzed interdemic competition with two populations of specialized predators against two prey which, in their turn, inhibit each other. The predator with a more uniform distribution captures more prey and achieves higher population density initially because it is more efficient. However, it increases the selective pressure on its prey creating an imbalance in its competition against other competitors of the same prey. This leads to exclusion of the prey, and then the predator itself is excluded because its preferred prey is no longer available. This fact facilitates the way of the most aggregated predator (simulations 1–4 in *2SpePred.json* in Supplementary Material). Such an effect is generally more applicable to any predator with higher efficiency (simulation 5 in *2SpePred.json* in Supplementary Material). Even the tandem predator/prey scenario with aggregated distribution strategies excludes any other tandem with more uniform strategies (simulation 3 in *2SpePred.json* in Supplementary Material). We must point out that these scenarios are only valid for specialized predators, which are highly adapted to capturing their preferred prey, such as the Eurasian lynx (*Lynx lynx*), specialized in small ungulates [102], or the coast horned lizard (*Phrynosoma coronatum*), specialized in local ants [103]. When predators are generalist they present other population dynamics because there is merely apparent competition [104,105]. We observe that two common prey of the predators remain in equilibrium despite one predator (*Strong*) being more efficient, due to a more uniform distribution (simulation 6 in *2SpePred.json* in Supplementary Material). Finally, the most efficient predator is predominant only in population density (Figure 9).

The host-parasite interaction constitutes a particular predation mode, which makes these results applicable to parasitism, and it is known that as long as parasites show enough variability within the host population, the host–parasite interaction can be stabilized [106,107], and the aggregation of parasites with regards to the hosts is a defining characteristic of the parasites’s metazoan population [108] and the pathogen’s ability to infect distant individuals in a spatially structured host population is known to lead to the evolution of a more virulent pathogen [109]. The habitat structure and the spatial scale play a central role in the predator–prey dynamic. These effects have been observed to operate in more complex groups of predators and preys and of multiple species [104,105]. In *ECA* we also see that this happens from a new perspective: the aggregate distribution prevails in the prey–predator interaction because its variability limits the effectiveness of the predator, and this means preserving the evolutionary balance of its prey and, therefore, of predators.

This ultimate goal of preserving the evolutionary balance of the interaction could be achieved in another way, as long as the effectiveness of the predator is decreased, for example, by decreasing the indirect offspring of the predator, passing the *IndirectOffpring* of its prey from 3 to 2 (simulation 5 in *2SpePred.json* in Supplementary Material). The result is similar to aggregated distribution: at first its population increases, but then the predator with the most offspring becomes extinct. It suggests that other mechanisms could produce the same effect, other than aggregated distribution. However, intraspecific competition wipes out any trait with lower fitness by natural selection, irrespective of whether it arose from mutation or variability. Besides, a less efficient population in terms of dispersion because conspecifics are grouped is, on the one hand, less costly adaptively speaking, as it only requires a gregarious instinct, and on the other, more profitable for the predator because it can increase its intraspecific competition, which will allow higher rates of survival and reproduction.

### 3.6. The Selfish Herd

There is an ongoing controversy as to whether life within a group benefits the individual who seeks protection or benefits the group as a *selfish herd* [71,110]. With spatial self-structuring in incomplete mixed media, positive selection of “altruistic” features becomes feasible [111]. From the strict viewpoint of dispersion strategies, as organism grouping implies aggregated distribution, the collective behavior only presents advantages for the group because it is more efficient with an aggregated distribution in the predator–prey interaction, whereas for the individual predator it is only costly. After all, the inefficiency of the aggregated distribution decreases the predator’s life expectancy as an individual. There is individual selection under this predator–prey system as long as only some predators can capture the necessary prey to reproduce. There is also multilevel selection as long as the groups with aggregated distribution can exclude other groups [112]. We conclude that we have a case of multilevel selection in which selective group pressure is higher than individual pressure. Therefore, the adaptive advantages for the group prosper in detriment to the individual [113] because any mutant with higher fitness cannot replace the original population, in the end, it would become extinct with the general population of predators, and a group of individuals with less fitness could replace another group with higher individual fitness.

The results obtained in *ECA* have corroborated ecological knowledge, which has been available for a long time, in a new way. Specifically, that the aggregate distribution of animals stabilizes the predator–prey interaction or that it promotes biodiversity. The method has also shown other obvious phenomena, such as the fact that aggregate distribution is more inefficient because animals that aggregate leave more untapped resources in patches that they do not occupy. However, it has provided a new perspective, considering the aggregate distribution as the closest source to achieving the final cause of the stability of the predator–prey interaction.

This work aims to present a theoretical advance by synthesizing previous theories, such as that of spatial heterogeneity or colonization-competence. Besides, it confirms the multilevel selection theory when applied to collective animal behavior. It can have practical implications for reintroducing endangered species and strategies to preserve biodiversity.

It would be advisable for future fieldworks to demonstrate the predictions based on the consideration that the most abundant plankton species are abundant because they are the most competent unless the *small portion* effect is powerful. Further work should search for predator–prey interactions that became extinct due to their uniform distribution (even from the fossil record).

The weakness of this study is inherent to all theoretical studies in evolutionary ecology, coupled with the limitation of being based partially on the existence of unstable interactions (such as specialized prey–predator interactions), which are difficult to trace due to their ephemeral ecological existence, despite having possibly left a trace of gregariousness.

## 4. Conclusions

In *ECA* we see how digital organisms distributed in an aggregate manner prevail and exclude others that are more uniformly distributed, but only when their interactions are of the predator–prey type. In this type of simulation, if prey and predators interact in a cell, only the predator has offspring, but if they do not interact, only the prey has offspring. With a uniform distribution, and due to the instability in this type of interaction, the predator, or the prey first and then the predator, can be excluded last. This instability is attenuated if both digital organisms are distributed in an aggregated manner. In other words, when they are aggregated, there is greater variability in the densities per cell, thereby increasing the probability that few predators will coincide with many prey in a cell. These cells with few predators constitute authentic adaptive shelters that increase the overall offspring of the prey and, therefore, of the predator. In ecological terms, to avoid extinction, both prey and predator give up competition capacity in exchange for colonization capacity.

In *ECA* we also see, from a new perspective and by the same mechanism, that the aggregate distribution of digital organisms presents an exception to the principle of competitive exclusion: by aggregating, digital species that inhibit each other do not get to exclude each other because, similar to the predator and its prey, they exchange competition capacity for colonization capacity.

In real systems, the instability of the predator–prey interaction has been documented with equations of the Lotka-Volterra type and with well-contrasted experimental studies, and it is also known that aggregation stabilizes this type of relationship. This long established knowledge in ecology has been verified in *ECA* but from a new perspective, where we see that animal aggregation can be understood as an interdemic adaptive mechanism, which is necessary to stabilize predator–prey interactions. This underlying mechanism of natural selection would explain why many prey and predators tend to aggregate, even without an apparent adaptive benefit, and how biodiversity thrives in marine environments, due to the aggregation of nekton and the permeability of the habitat that favors the aggregation of plankton.

## Data Availability

Not applicable.

## Supplementary Materials

The following supporting information can be downloaded at: https://www.mdpi.com/article/10.3390/biology11101477/s1 and https://github.com/juanfal/AE4 accessed on 15 July 2022.
